# Dental Erosion and the Gouty Diathesis

**Published:** 1892-12

**Authors:** E. T. Darby


ARTICLE VI.

### DENTAL EROSION AND THE GOUTY DIATHESIS.

All who have studied the subject of erosion admit that there is a mystery which they cannot fathom; and yet there is a general belief that it is the result of an acid condition of the fluids of the mouth, more especially the product of the labial and buccal mucous glands. Granting that this be so, there must be some exciting cause which alters the character of these secretions, making them more than normally acid in their reaction, for the usual tests with litmus paper reveal a slight acid reaction in the mouths of nearly all persons, especially upon arising in the morning; and yet erosion is the exception, and not the rule.

That the exciting cause is a local one, the result of an irritated condition of the glands themselves, I cannot believe. That it is a constitutional derangement of the functions of the larger organs of the body, which so alters the character of the blood-serum, I do believe.

The humoral pathologists, from Hippocrates to Garrod, believed that the cause of gout was to be looked for in the perverted physiological functions of the fluids of the body. Most modern pathologists are accepting the uric-acid theory of gout, as promulgated by Murray, Forbes and Wolloston; while still others believe, with Cullen, that gout is a trophoneurosis. Whatever the cause may be, the manifestations are so protean that there may never be a perfect harmony of belief as to its origin. One fact is well established; that there is in the gouty subject a decidedly acid reaction to all the secretions of the body. Whether this pathological condition is sufficient to produce the changes which we recognize in the oral fluids, I am unable to say. However, that there are remarkable coincidences, existing between the two diseases, there can be no doubt. Erosion, like gout, is a disease of advanced civilization. I have never seen it during the period of growth and development, when the processes of nutrition are active, and the consumption of food in excessive quantities is rendered possible by the large demands for the needs of the growing body and for the development of active energy. It is common in adult life, when the processes of nutrition are less active and when growth is complete, and when the supply of food needs to be regulated according to the amount of energy to be developed. Erosion, like gout, is more frequently than otherwise found in the person of those who live in luxury and refinement, and in an atmosphere where an abundant supply of oxygen is wanting. I do not remember to have ever seen a case of erosion in the mouth of one who earned his daily bread by the sweat of his brow. If, then, erosion be a constitutional disease, and in any way associated with the gouty diathesis, none but constitutional treatment will avail. I think it must be admitted that all efforts to arrest its ravages by local measures have resulted in failure. The heroic method of inserting gold fillings upon the eroded surface has done more than anything else to arrest its progress; and yet I feel sure that your own experience has taught you that in some instances this has been ineffectual. The ingenious device of Dr. Kells, of having the patient wear a rubber splint to protect the eroded surfaces from the action of the acid mucus during the night, or when the mouth is in repose, may aid in retarding the disease, but it cannot wholly arrest it.

It would seem that the most logical line of treatment is in the direction of primarily improving the digestion, by the avoidance of such articles of food as experience has demonstrated are incompatible with the gouty dyscrasia. If there is one signal peculiarity in the digestive arrangement of gouty persons, it is their limited power to digest the carbohydrates, the sugars and starches. In whatever form these foods are used, they are more commonly the source of the dyspeptic troubles of sufferers from gout than are the albuminous foods. They provoke the acid and flatulent dyspepsia which so generally precedes the explosion of the gouty paroxysm. Abstinence from all the fermented preparations of alcohol is one of the most important restrictions, on account of the unfermented dextrine and sugar which they contain. Experience has shown that the prohibition of some of the sub-acid fruits—such as, for example, strawberries, apples, watermelons, and grapes—is attended with beneficial results. Next in order to the saccharine foods as the source of indigestion in gouty persons, come the amylaceous aliments. These constitute necessarily so large an element in ordinary diet that the limitation of them in the dietary of gouty persons applies, in the majority of cases, only to their excessive use. The purely starchy aliments, such as potatoes and the preparations of corn and rice, and even those which contain a considerable portion of gluten, like wheat, oatmeal, and barley, often provoke in gouty subjects a great deal of mischievous and painful indigestion.

"Next in importance to diet as a hygienic regulation in the management of gouty patients, is enforced exercise.

The axiom of Abernethy, 'To live on a shilling a day, and earn it,' comprises the philosophy of the true relations of food to work, and both to the highest development of physical health. Exercise is to be enforced, not simply as the means of securing an active respiration, and thereby an abundant supply of oxygen, but also as a means of converting the potential energy of the food consumed into vital energy. The essential condition, moreover, of healthy nutrition in every organ and in every tissue, is the maintenance of a vigorous functional activity. Hence the question of exercise in its largest sense involves not only muscular work, but work of every kind which tends to promote a healthy activity of the psychical as well as the physical functions. Muscular exercise in the open air has a special value for the victims of the gouty dyscrasia, by equalizing the circulation, quickening the respiratory movements, and stimulating the elimination of effete matters from the skin and lungs. But mental work and wholesome diversions are not less important, as antagonizing the evil effects of indolence and over-feeding, which are among the common predisposing causes of acquired gout."—PROF. E. T. DARBY, *Dental Cosmos.*

